# Chest magnetic resonance imaging: a protocol suggestion*

**DOI:** 10.1590/0100-3984.2014.0017

**Published:** 2015

**Authors:** Bruno Hochhegger, Vinícius Valério Silveira de Souza, Edson Marchiori, Klaus Loureiro Irion, Arthur Soares Souza Jr., Jorge Elias Junior, Rosana Souza Rodrigues, Miriam Menna Barreto, Dante Luiz Escuissato, Alexandre Dias Mançano, César Augusto Araujo Neto, Marcos Duarte Guimarães, Carlos Schuler Nin, Marcel Koenigkam Santos, Jorge Luiz Pereira e Silva

**Affiliations:** 1 PhD, Associate Professor, Universidade Federal de Ciências da Saúde de Porto Alegre (UFCSPA), Porto Alegre, RS, Brazil.; 2 MD, Resident in Radiology and Imaging Diagnosis, Irmandade Santa Casa de Misericórdia de Porto Alegre, Porto Alegre, RS, Brazil.; 3 PhD, Full Professor Emeritus, Universidade Federal Fluminense (UFF), Niterói, RJ, Brazil.; 4 PhD, Consultant Radiologist, Liverpool Heart and Chest Hospital NHS Trust, Liverpool, UK.; 5 PhD, Professor, Faculdade de Medicina de São José do Rio Preto (Famerp), São José do Rio Preto, SP, Brazil.; 6 PhD, Associate Professor, Centro de Ciências das Imagens e Física Médica (CCIFM) - Faculdade de Medicina de Ribeirão Preto da Universidade de São Paulo (FMRP-USP), Ribeirão Preto, SP, Brazil.; 7 PhD, Professor, Program of Post-graduation in Radiology, Universidade Federal do Rio de Janeiro (UFRJ), Rio de Janeiro, RJ, Brazil.; 8 PhD, Associate Professor, Department of Medical Practice, Universidade Federal do Paraná (UFPR), Curitiba, PR, Brazil.; 9 MD, Radiologist, Radiologia Anchieta, Taguatinga, DF, Brazil.; 10 PhD, Associate Professor, Universidade Federal da Bahia (UFBA), Salvador, BA, Brazil.; 11 PhD, Professor, Program of Post-graduation stricto sensu, A.C.Camargo Cancer Center, São Paulo, SP, Brazil.; 12 PhD, Attending Physician at Hospital das Clínicas da Faculdade de Medicina de Ribeirão Preto da Universidade de São Paulo (HCFMRP-USP), Ribeirão Preto, SP, Brazil.; 13 PhD, Associate Professor, Department of Medicine and Diagnostic Support, Universidade Federal da Bahia (UFBA), Salvador, BA, Brazil.

**Keywords:** Magnetic resonance imaging, Lung, Chest, Protocol, Sequences

## Abstract

In the recent years, with the development of ultrafast sequences, magnetic resonance imaging (MRI) has been established as a valuable diagnostic modality in body imaging. Because of improvements in speed and image quality, MRI is now ready for routine clinical use also in the study of pulmonary diseases. The main advantage of MRI of the lungs is its unique combination of morphological and functional assessment in a single imaging session. In this article, the authors review most technical aspects and suggest a protocol for performing chest MRI. The authors also describe the three major clinical indications for MRI of the lungs: staging of lung tumors; evaluation of pulmonary vascular diseases; and investigation of pulmonary abnormalities in patients who should not be exposed to radiation.

## INTRODUCTION

Pulmonary parenchyma imaging represents a unique challenge for magnetic resonance imaging (MRI). Limited signal intensity is caused by low proton density, susceptibility artifacts are due to differences between tissue and air, besides physiological motion (cardiac pulsation, respiration). Recently, further improvements in MRI techniques have widened the potential for investigation of pulmonary parenchymal diseases. Such techniques include very short echo times, ultrafast turbo-spin-echo acquisitions, projection reconstruction technique, breathhold imaging, and recently developed contrast agents (for perfusion and ventilation imaging)^(1-3)^.

In healthy lungs, the tissue density is 0.1 g/cm^3^, which is about tenfold lower than in other soft tissue organs. As the MRI signal intensity is directly proportional to the tissue proton density, even under perfect imaging conditions (i.e. neglecting relaxation effects), the MRI signal from the lung is ten-times weaker than that from adjacent tissues. The low signal-to-noise ratio makes proton MRI of the lung microstructure challenging. Signal averaging can be employed to increase the signal-to-noise ratio, but this extends the image acquisition times beyond 10 min per data set, which would make the protocols unsuitable for clinical routine. The signal-to-noise ratio may be increased with larger voxel sizes; however, smaller lesions such as peripheral lung metastases might not be visible due to partial volume effects^(1-3)^.

To obtain broad clinical acceptance, MRI of the lung has to be practical, robust, and reproducible. On top of these workflow aspects, MRI should provide consistently high image quality as well as diagnostic accuracy and a positive therapeutic impact. Different scanner manufacturers already provide the necessary sequences to perform MRI of the lung^(1-3)^. In the present article, the authors review the clinical and technical aspects of the method and suggest a protocol to be used in clinical routine for chest MRI.

## CLINICAL INDICATIONS FOR MRI OF THE LUNG

### Detection and characterization of pulmonary nodules

**Figure 1:**
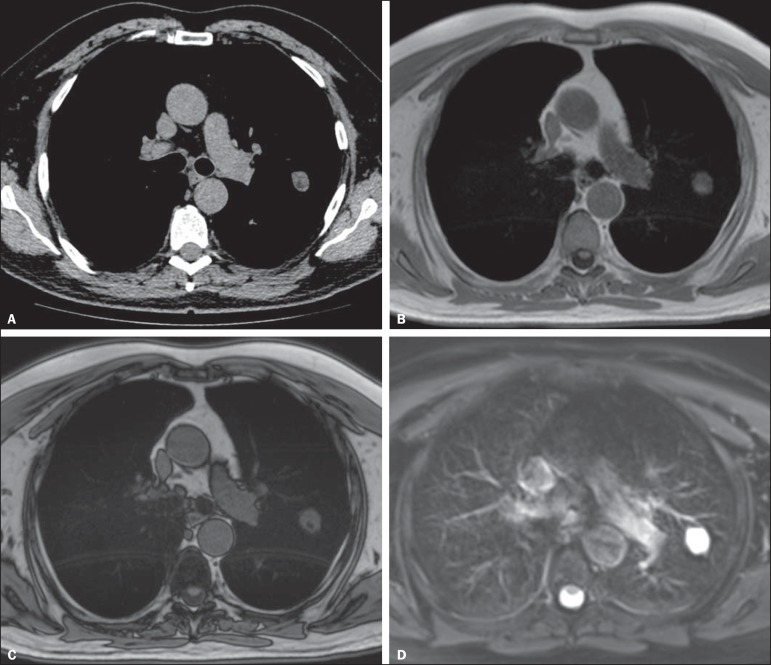
A 56-year-old patient with pancreatic cancer. **A:** Axial CT image showing low attenuation areas inside the nodule. This nodule had mean -33 HU attenuation. In-phase (**B**) and opposed-phase (**C**) images showing signal loss in the nodule, suggesting hamartoma. **D:** T2-weighted sequence showing high signal intensity of the nodule.

A recent meta-analysis reported that dynamic computed tomography (CT) and MRI, both of which are noninvasive methods, are equally accurate in distinguishing between malignant and benign solitary pulmonary nodules, and the differences between the two methods are insignificant^(4)^. The authors of the meta-analysis have found that, for the 10 dynamic CT studies, MRI had a pooled sensitivity of 93% (95% CI: 0.88-0.97) and a pooled specificity of 76% (95% CI: 0.68-0.97)^(5)^. Koyama et al.^(4)^ have reported that non-contrast-enhanced MRI of the lung is as efficient as is thin-section multidetector CT in detecting malignant nodules. The authors have also found that the overall nodule detection rate at each MRI sequence (82.5%) was significantly lower than was that for multidetector CT (97.0%), although there was no significant difference between the two techniques in terms of malignant nodules detection rate. Also, chemical shift MRI has been described to detect fat in pulmonary hamartomas^(5)^ (Figure 1). Diffusion-weighted MRI may be able to be used in place of ^18^F-fluorodeoxyglucose positron emission tomography (FDG-PET) to distinguish malignant from benign pulmonary nodules/masses with fewer false-positive results as compared with FDG-PET^(6)^.

### Tumor-node-metastasis staging

**Figure 2:**
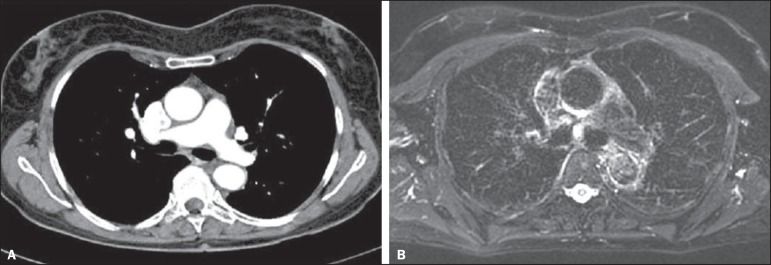
A: Axial CT image showing a 8 mm lymph node in the subcarinal station. **B:** Axial T2-weighted image with fat saturation showing a high signal intensity in this lymph node, suggesting metastatic disease.

In the tumor-node-metastasis (TNM) staging system, the T stage (tumor size and invasion degree) is the primary determinant of the neoplasia severity^(7)^. MRI is superior to CT for depicting the pericardium, heart, and mediastinal vessels and, therefore, it can be indicated in specific situations such as superior vena cava obstruction, myocardial invasion, or tumor spread into the left atrium via pulmonary veins^(8)^. In addition, MRI allows for lung cancer to be distinguished from secondary changes due to atelectasis or pneumonitis^(7)^. At T2-weighted images, post-obstructive atelectasis and pneumonitis often show higher signal intensity when compared with the central tumor^(7)^. Ohno et al.^(9)^ have conducted a prospective study of 115 consecutive lung cancer patients submitted to CT, short-tau inversion-recovery turbo spin-echo (STIR-TSE) imaging (Figure 2), and FDG-PET/CT, as well as surgical and pathological examination. The authors have found that, on a per-patient basis, the quantitative sensitivity and accuracy of STIR-TSE imaging (90.1% and 92.2%, respectively) were significantly higher than were the quantitative sensitivity, qualitative sensitivity, quantitative accuracy, and qualitative accuracy of co-registered FDG-PET/CT (76.7%, 74.4%, 83.5%, and 82.6%, respectively). Previous studies have demonstrated that whole-body MRI provides acceptable accuracy, and that its efficacy in lung cancer staging is comparable to that of PET/CT^(8,9)^. Each of those two imaging modalities has been shown to have its advantages^(7)^, whole-body MRI being superior in the detection of brain and liver metastases, while PET/CT performs better for detecting lymph node and soft-tissue metastases. Diffusion-weighted MRI is a promising technique to evaluate areas that have been submitted to radiation therapy^(7)^. Whole-body diffusion-weighted MRI can be used for the assessment of the M (metastasis) stage in non-small cell lung cancer patients and has been shown to be as accurate as is PET/CT^(9)^.

### Pulmonary thromboembolic disease

**Figure 3:**
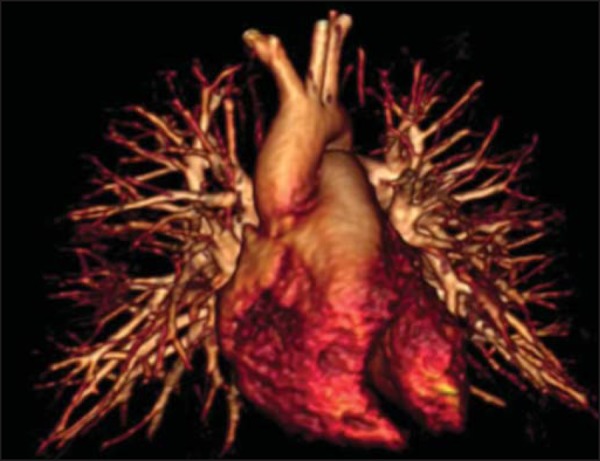
3D volume rendering of MR angiography showing subsegmental resolution.

Pulmonary embolism is the third most common cause of acute cardiovascular disease (after myocardial infarction and stroke). It frequently goes undetected and is therefore responsible for thousands of deaths every year^(10)^. In 2003, Stein et al.^(11)^ conducted a meta-analysis of the use of gadolinium-enhanced MRI to depict acute pulmonary embolism. The authors used conventional pulmonary angiography as the reference standard. They found that the reported procedure sensitivity covered a broad range (77-100%) and that the reported specificity was uniformly high (95-98%)^(11)^ (Figure 3). In the most recent of the studies evaluated in this meta-analysis, Oudkerk et al.^(12)^ showed that the sensitivity of contrast-enhanced MRI for pulmonary embolism was 100% in central and lobar arteries, 84% in segmental arteries, and only 40% in subsegmental branches. Overall, the combined MRI protocol has been found to be more reliable and sensitive than is 16-slice multidetector CT^(13)^. The average MRI examination time is reported to be approximately 10 min^(13)^.

### Pulmonary hypertension

The use of MRI allows a comprehensive assessment of pulmonary hypertension; especially when performing MR angiography and perfusion MRI, it is possible to differentiate between chronic thromboembolic pulmonary hypertension and pulmonary arterial hypertension^(14,15)^. In addition, MR angiography allows for an in-depth evaluation of the thromboembolic material location, and, for surgical planning, is equally as useful as are digital subtraction angiography and CT angiography^(16,17)^. In MRI, perfusion sequences can be quantitatively evaluated, allowing for assessing the severity of small-vessel disease. Structural imaging of the lung will allow ruling out parenchymal diseases. Measurements of blood flow and right heart pressure allow pulmonary arterial pressure and cardiac strain to be estimated, besides facilitating the identification of concomitant valvular disease^(17)^.

### Patients with cystic fibrosis

**Figure 4:**
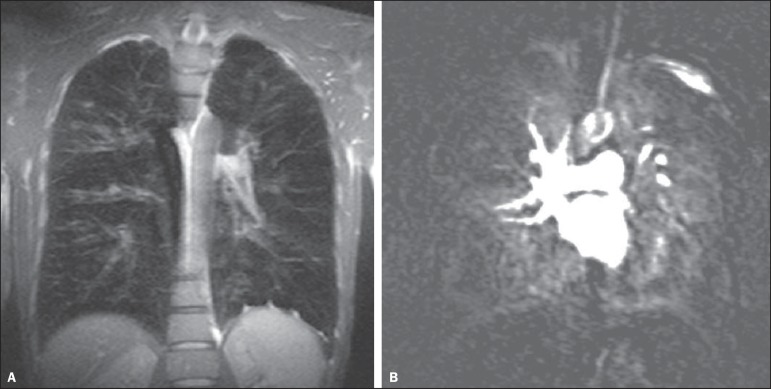
32 year-old patient with cystic fibrosis. **A:** Coronal T1-weighted gradient echo sequence (VIBE) with 2 mm slice thickness. Note the presence of bronchiectasis with mucoid impaction. **B:** Pulmonary perfusion shows multiple perfusion defects better characterizing the disease severity.

The standard radiological tools for monitoring lung disease in patients who have cystic fibrosis are chest X-ray and high resolution CT (HRCT), and for this purpose different scoring systems are proposed^(18)^. Scans obtained using thin sections provide images of lung structure with micrometer-scale resolution, and HRCT and/or MRI findings can also constitute useful outcome measures for studies of lung disease in cystic fibrosis patients^(19,20)^. Additionally, MRI can be used to assess various aspects of pulmonary function, including lung perfusion (Figure 4)^(21,22)^, blood flow^(23)^, respiratory mechanics^(24,25)^, and (with the administration of inhaled contrast agents) pulmonary ventilation^(26)^. Recent studies have shown that MRI is highly capable of revealing the bronchiectasis typical of cystic fibrosis, as well as mucus plugging, and that MRI has a diagnostic value equal to that of CT in grading the severity of the disease with the Bhalla or Helbich score^(27)^.

### Patients with pneumonia

**Figure 5:**
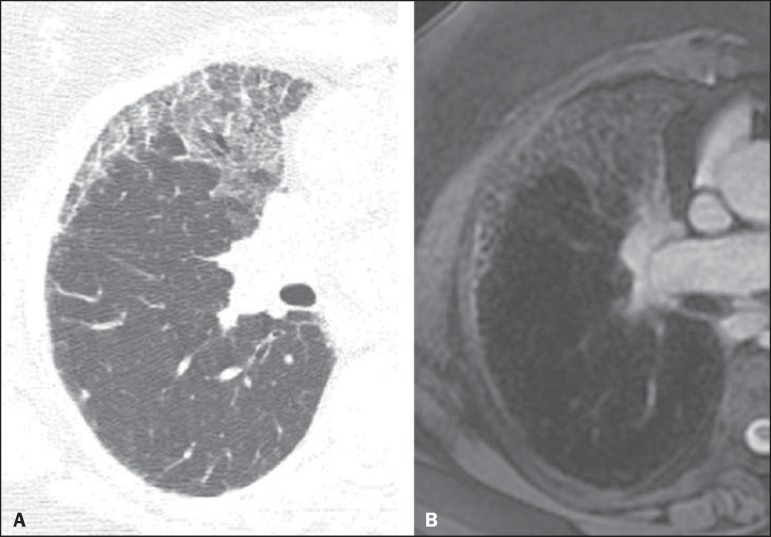
**A:** Axial HRCT image showing homogeneous, segmental ground glass opacities in the pulmonary cortex. **B:** Axial T2-weighted image clearly demonstrates the lesion.

The various features of pneumonia, such as ill-defined nodules, ground-glass opacities, and consolidations can be easily detected and differentiated by MRI (Figure 5). At chest MRI, extremely small opacities and calcifications represent great challenges because of the thicker slices and the low signal intensity. As a follow-up tool, MRI is recommended over CT, in order to avoid excessive exposure to ionizing radiation. The sensitivity of T2-weighted sequences and the potential of contrast-enhanced T1-weighted sequences can greatly facilitate the differential diagnosis^(28)^. In addition, incipient complications such as pericardial effusion, pleural effusion, empyema, and lung abscess are easily recognized on MRI scans^(28)^. In immunocompromised patients, MRI is nearly as accurate as is CT for detecting pulmonary abnormalities associated with infection^(29-31)^.

## TECHNICAL ASPECTS AND PROTOCOL SUGGESTION

For MRI of the lung, standard scanners with field strength of 1.5 tesla (T) and full parallel imaging capabilities are recommended^(2,3,32)^. Although higher field strength, i.e., 3 T, will theoretically increase the signal-to-noise ratio, faster signal decay caused by susceptibility artifacts poses additional obstacles to lung imaging. Special efforts are needed to obtain similar results at 3 T as compared with 1.5 T, e.g., when imaging nodules^(33)^.

Oxygen in air is paramagnetic, and tissue is diamagnetic, which leads to a bulk magnetic susceptibility difference (Δ_χ_ = 8 ppm) at lung-air interfaces. At each tissue interface, the susceptibility difference forms a static local field gradient. The multiple microscopic surfaces presented by the airways and alveoli in the lungs thus create highly inhomogeneous local magnetic field gradients on a spatial scale smaller than the size of a typical imaging voxel (2-5 mm)^(1-3)^. These microscopic field gradients lead to a rapid dephasing in gradient echo imaging; this signal decay is typically described by an apparent transverse relaxation time T2*, which can be as short as 2 ms or less at B0 = 1.5 T. Thus, gradient echo MRI of lung parenchyma becomes highly challenging and requires pulse sequences with short echo times (TE < 1-2 ms)^(1-3)^. As the magnetic field inhomogeneity increases with B0, even shorter T2* of about 0.5 ms are found at 3 T. Frequently, the expected signal-to-nose ratio gain of 3 T over 1.5 T cannot be realized, because it requires that TE is shortened accordingly. With identical pulse sequences, a shorter TE can only be achieved if more powerful gradient systems are used, but current 3 T MRI systems often utilize the same gradient units as high-end 1.5 T systems.

A basic protocol is mainly based on non-contrast breath-hold sequences and free breathing diffusion-weighted imaging (Table 1). During this time, three-dimensional (3D) T1-weighted gradient echo and T2-weighted fast spin echo as well as STIR sequences can be used. Respiratory, vascular and cardiac motion can be addressed by fast imaging, gating, and triggering techniques, respectively (Figure 6 - A-F)^(34)^.

**Table 1:** Basic protocol

GE	Siemens	Slice thickness
Axial, T2-weighted sequence FSE Fiesta	T2-weighted sequence FSE TrueFisp	5.0 mm
Coronal, T2-weighted sequence FSE Fiesta	T2-weighted sequence FSE TrueFisp	5.0 mm
Axial, T2-weighted sequence FSE with fat suppression	Axial, T2-weighted sequence Blade axial	5.0 mm
Axial diffusion-weighted	Axial diffusion-weighted	5.0 mm
		B0 and B600
T1-weighted sequence LAVA with fat suppression	T1-weighted sequence VIBE with fat suppression	2.5 mm
T1-weighted sequence LAVA without fat suppression	T1-weighted sequence VIBE without fat suppression	2.5 mm
T1-weighted sequence in- and out-of-phase	T1-weighted sequence in- and out-of-phase	5.0 mm
Coronal, T2-weighted sequence HASTE	T2-weighted sequence HASTE coronal	5.0 mm
Perfusion MRI T1-weighted sequence	Perfusion MRI T1-weighted sequence	20-40 acquisition phases
T1-weighted sequence LAVA with fat suppression	T1-weighted sequence VIBE with fat suppression	60 s after injection

Figure 6

**Figure:**
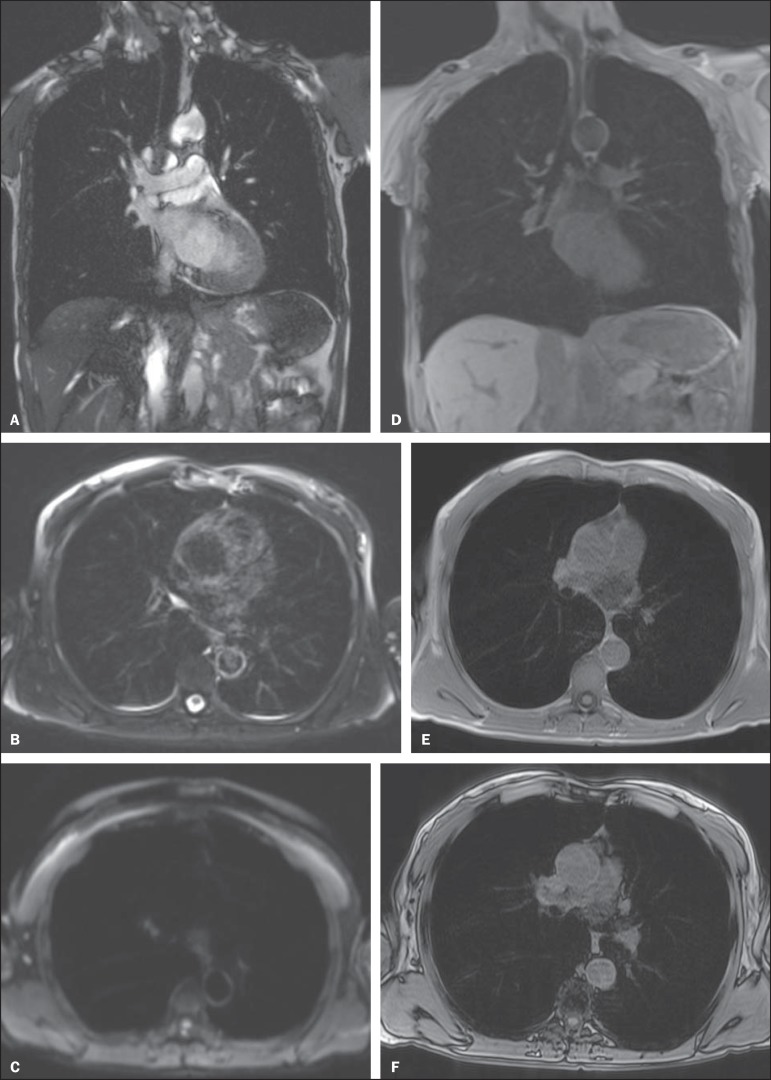
**(A-F)**. Image examples for the suggested protocol. **A:** Coronal T2-weighted FSE TrueFisp sequence (4 mm). **B:** Axial, T2-weighted sequence Blade axial (4 mm). **C:** Axial, diffusion-weighted image (5 mm). **D:** Coronal, T1-weighted sequence VIBE with fat suppression (4 mm). **E,F:** T1-weighted sequence in- and out-of-phase.

**Figure:**
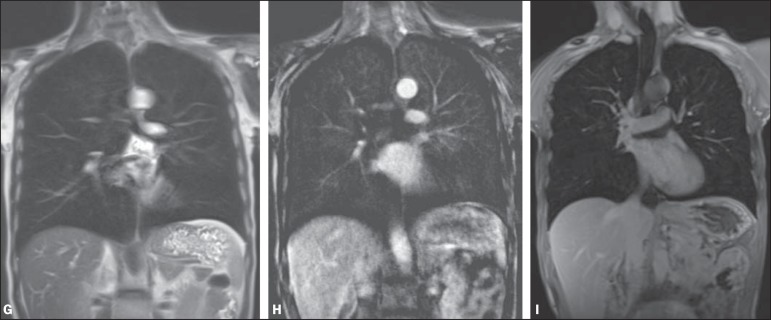
**(G–I):** Image examples for the suggested protocol. **G:** Coronal, T2-weighted HASTE sequence. **H:** Coronal, T1-weighted perfusion sequence. **I:** T1-weighted sequence VIBE with fat suppression.


Half-Fourier acquisition and ultra-short echo times are recommended^(32)^. The basic protocol should be extended to contrast-enhanced imaging with high spatial resolution (single-phase MR angiography) or high temporal resolution as in time-resolved perfusion imaging (Figure 6 - G,H). Complicated and time-consuming sequences requiring respiratory or cardiac gating should be reserved for specific clinical scenarios. T1-weighted 3D gradient echo sequences such as a volume interpolated breath-hold examination (VIBE) are recommended for the assessment of the mediastinum, pulmonary nodules, masses and consolidations, and should be repeated with fat saturation after the administration of contrast material. In chronic obstructive pulmonary disease, for example, the contrast agent compensates for the decreased signal intensity due to the properties of the "minus-pathology"^(32)^ (Figure 6 - I). A T2-weighted fast spin echo half-Fourier acquisition sequence will easily visualize pulmonary infiltrates, inflammatory bronchial wall thickening, as well as mucus and fluid accumulation. Experimental work has shown that the sensitivity of T2-weighted breath-hold or respiratory-gated sequences for infiltrates at least equals that of chest X-ray and multidetector CT^(13)^.

The use of diffusion-weighted sequences in the assessment of masses or lymph node involvement is still subject to evaluation, but has also shown promising results for whole-body staging of lung cancer^(34-36)^. However, whole-body MRI with modern fast techniques has been suggested as a diagnostic tool for M staging, which can be implemented into a comprehensive lung cancer staging protocol. Such a technique has already proved to be sensitive to detect extrathoracic spread of lung cancer. A direct comparison between whole-body MRI and FDG-PET/CT has shown that whole-body MRI has significantly higher sensitivity for detecting metastatic disease mainly due to a higher accuracy in determining the degree of brain, neck and bone involvement^(37-39)^.

Contrast-enhanced perfusion MRI is a straightforward and easy-to-implement technique. Three-dimensional T1-weighted gradient echo sequences with the use of parallel imaging and echo sharing allow for short acquisition times of approximately 1.5 s for a 3D dataset (so-called 4D or 3D + t) required to visualize perfusion during the peak enhancement of the lung parenchyma.

## CONCLUSION

With its rapid development in recent years, MRI of the lung is now truly at the threshold of broad clinical application. Disease of the airways and vasculature, as well as nodules and lung cancer, now constitute a major focus. Because MRI offers a number of advantages when compared with conventional nuclear medicine techniques, it is competing with multidetector CT in many applications. The fact that it does not involve the use of ionizing radiation puts chest MRI in a front-line position for all cross-sectional imaging studies, particularly in cases involving young patients. The unique combination of structural and functional information makes MRI attractive for use in all diseases where the choices among innovative and expensive treatment options will actually require and benefit from an increased number of measurable parameters.
